# Comprehensive evaluation of inactivated SARS-CoV-2 vaccination on sperm parameters and sex hormones

**DOI:** 10.3389/fimmu.2024.1321406

**Published:** 2024-02-26

**Authors:** Yehao Dong, Zaihua Ba, Yining Qin, Jiao Ma, Yuqi Li, Yingze Zhang, Aijun Yang, Fei Chen

**Affiliations:** ^1^ Center for Reproductive Medicine, Affiliated Hospital of Jining Medical University, Jining, China; ^2^ Department of Physiology, Jining Medical University, Jining, China

**Keywords:** inactivated SARS-CoV-2 vaccine, semen parameters, sex hormones, China, SARS-CoV-2, COVID-19, male fertility

## Abstract

**Background:**

The inactivated severe acute respiratory syndrome coronavirus 2 (SARS-CoV-2) vaccine has made significant contributions to fighting the epidemic in the past three years. However, the rapid development and application raised concerns about its safety in reproductive health, especially after several studies had observed a decrease in semen parameters following two doses of mRNA SARS-CoV-2 vaccination. Thus, it is necessary to comprehensively evaluate the effect of inactivated SARS-CoV-2 vaccine on male fertility.

**Methods:**

A retrospective cohort study was conducted in the Center for Reproductive Medicine of the Affiliated Hospital of Jining Medical University between July 2021 and March 2023. A total of 409 men with different vaccination status and no history of SARS-CoV-2 infection were included in this study. Their sex hormone levels and semen parameters were evaluated and compared separately.

**Results:**

The levels of FSH and PRL in one-dose vaccinated group were higher than other groups, while there were no significant changes in other sex hormone levels between the control and inactivated SARS-CoV-2 vaccinated groups. Most semen parameters such as volume, sperm concentration, total sperm count, progressive motility and normal forms were similar before and after vaccination with any single dose or combination of doses (all *P* > 0.05). Nevertheless, the total motility was significantly decreased after receiving the 1 + 2 doses of vaccine compared to before vaccination (46.90 ± 2.40% *vs*. 58.62 ± 2.51%; *P* = 0.001). Fortunately, this parameter was still within the normal range.

**Conclusion:**

Our study demonstrated that any single dose or different combined doses of inactivated SARS-CoV-2 vaccination was not detrimental to male fertility. This information could reassure men who want to conceive after vaccination and be incorporated into future fertility recommendations.

## 1 Introduction

The coronavirus disease 2019 (COVID-19) pandemic, caused by the severe acute respiratory syndrome coronavirus 2 (SARS-CoV-2) infection, has triggered a worldwide health emergency of an unparalleled scale. As of January 2024, over 774 million infected cases and 7.0 million fatalities have been recorded globally (1). In most healthy adults and children, the disease is usually mild or asymptomatic. Common clinical symptoms include fever, cough, myalgia or fatigue, expectoration, and dyspnea (2). However, the illness may be severe and even fatal in some patients, especially the elderly and those with underlying diseases (3). As well known, before a highly specific drug is developed, large-scale vaccination is the important approach to mitigate disease and build herd immunity against this pandemic. By 26 November 2023, a total of 13.59 billion vaccine doses have been administered (1), and they successfully reduced the rates of infections, severity, hospitalization and mortality among the different populations (4). Even for XBB.1.5, the most recent Omicron subvariant, vaccination still confers some levels of protection against it, especially against severe disease (5–7). Currently, there are more than 183 vaccine candidates in clinical trials, and 14 of them have been granted emergency use authorization (8). Among them, Sinovac/CoronaVac and Sinopharm BBIBP-CorV are predominantly used in China and approved in other 114 countries such as Brazil, Chile, Indonesia, Mexico, and Turkey (9). Both of them are inactivated vaccines, which administer the virus in a chemically inactivated form that is unable to replicate. The main antigenic components that induce immune responses are retained, and additionally, aluminum hydroxide is added as an adjuvant to strengthen the immune response. Although the neutralizing antibody titres in individuals who vaccinated with inactivated vaccine were lower than with mRNA vaccine (10), the efficacy of CoronaVac in preventing the need for assistance (defined as a score ≥3 on the WHO Clinical Progression Scale) could still be as high as 83.7% (11). Similarly, in a randomized clinical trial involving 40,382 participants, vaccination of adults with Sinopharm BBIBP and WIBP vaccines markedly decreased the risk of symptomatic COVID-19, and serious adverse events were rare (12). Moreover, because of their long shelf life without the need for ultracold chain storage, inactivated vaccine has been proposed to be a near-ideal candidate for mass immunization programs in less wealthy nations (12, 13).

With the implementation of the inactivated COVID-19 vaccination, questions regarding the impact of the vaccine on male reproductive health have arisen. In this regard, Zhu et al. observed that there were no significant differences in semen parameters (volume, sperm concentration, progressive motility and total progressive motile count) before and after receiving first or second dose of the vaccine (14). Our previous study also showed similar results (15). However, these studies focused on evaluating the effect of each individual dose vaccination, while the influence of combined doses of vaccines on human fertility has not been assessed in detail. Although some studies have found a temporary decrease in sperm concentration and total sperm motility after 1 + 2 doses of mRNA vaccination compared to unvaccinated men, whether this conclusion applies to inactivated vaccines remains to be verified (16, 17). Moreover, most of the participants included in previous studies on inactivated vaccines underwent semen analysis only once, making it difficult to use a pre and post control group design to eliminate confounding factors and selection bias (15, 18, 19). Therefore, we performed this study to comprehensively investigate the effect of inactivated COVID-19 vaccination on male fertility, hoping to provide more valuable information for future public health efforts.

## 2 Materials and methods

### 2.1 Study design and setting

This is a retrospective cohort study conducted in the Center for Reproductive Medicine of the Affiliated Hospital of Jining Medical University between July 2021 and March 2023. The study protocol obtained approval from the Ethics Committee.

### 2.2 Participant selection

The study included 409 men aged between 22 and 49 years who had attended the center for reproductive problems or routine examination. Men who were tested positive for SARS-CoV-2 at any point or were taking medications such as supplemental testosterone or anabolic steroids were excluded.

Two sets of comparative observations were conducted simultaneously. One was the comparison of sex hormone levels before and after inactivated SARS-CoV-2 vaccination. Another was to evaluate the effect of each dose or combination of doses of vaccination on semen parameters. Based on their vaccination status, subjects participating in the first comparison were categorized into four groups: unvaccinated group (group A, n = 110), one-dose vaccinated group (group B, n = 11), fully vaccinated group (group C, n = 156), and booster group (group D, n = 106). In the second evaluation, participants had at least two semen analyses and were categorized into six groups according to the time point of each analysis: before and after the first dose of vaccine (group A1, n = 3), before and after the second dose of vaccine (group B1, n = 10), before and after the booster dose of vaccine (group C1, n = 40), before and after 1 + 2 doses of vaccine (group D1, n = 56), before and after 2 + 3 doses of vaccine (group E1, n = 7), before and after 1 + 2 + 3 doses of vaccine (group F1, n = 28).

Sex hormonal analysis: Fasting blood samples were obtained from participants for sex hormonal analysis. Serum levels of follicle-stimulating hormone (FSH), estradiol (E2), prolactin (PRL), testosterone (T) and luteinizing hormone (LH) were determined via the Unicel Dxi 800 Access Immunoassay System (Beckman Coulter Inc.). At the same time, we also calculated T/LH ratio, which is a valuable indicator of Leydig cell function (20, 21).

Semen analysis: Following a 2–7-day abstinence period, each subject provided a fresh semen sample in a sterile vessel by means of masturbation. The samples were liquefied at room temperature for 30–60 min and then evaluated according to Laboratory Manual for the Examination and Processing of Human Sperm (5th Edition) (22). Semen volume and morphology were determined with the help of graduated tube and Diff Quick Staining Kit (Huakang Biomed Ltd.), respectively. Other parameters included sperm concentration, total sperm count, progressive motility and total motility were analyzed by computer-assisted sperm analysis system (SAS Medical Co., Ltd.).

### 2.3 Data collection

The baseline (age, height, and weight) and clinical data were extracted from the patients’ medical record. Other details including vaccination date and type of vaccine administered were gathered through interviews.

### 2.4 Data analysis

In this retrospective cohort study, SPSS 27.0 (SPSS Inc.) was used for all statistical analysis. For continuous variables, the data were represented as mean ± standard error (SE) and compared by one-way analysis of variance and Duncan’s multiple range test. Categorical variables were expressed as numbers (n) and percentages (%) of the total and analyzed using χ^2^ test or the Fisher exact test (samples with expectancy of less than 5). Differences among groups were considered as statistically significant when *P* < 0.05.

## 3 Results

The sociodemographic characteristics and sex hormone levels of 383 subjects were described in **Table 1**. There were no statistically significant differences in age (*P* = 0.056) and BMI (*P* = 0.250) between the control and inactivated SARS-CoV-2 vaccinated groups. Most subjects were healthy without a history of testicular surgery. Pernicious habits such as smoking and alcohol abuse were rare.

**Table 1:** Sociodemographic characteristics of the men enrolled in the study and sex hormone levels before and after vaccination.

Parameter	Group A (n = 110)	Group B (n = 11)	Group C (n = 156)	Group D (n = 106)	*P* value	Statistical test
	Unvaccinated group	One-dose vaccinated group	Fully vaccinated group	Booster group		
Participants’ characteristics						
Age (years), mean ± SE	31.26 ± 0.46^a^	33.27 ± 1.48^a^	33.03 ± 0.40^a^	33.81 ± 0.53^a^	0.056	one-way ANOVA test
BMI (kg/m^2^), mean ± SE	26.85 ± 0.40^a^	25.91 ± 1.36^a^	26.65 ± 0.34^a^	25.62 ± 0.34^a^	0.250	one-way ANOVA test
Smoking % (n/total)	6.36 (7/110)^a^	9.09 (1/11)^a^	7.05 (11/156)^a^	11.32 (12/106)^a^	0.473	Fisher exact test
Alcohol consumption % (n/total)	0 (0/110)^a^	0 (0/11)^a^	0 (0/156)^a^	0.94 (1/106)^a^	0.305	Fisher exact test
Testes surgery % (n/total)	0.91 (1/110)^a^	0 (0/11)^a^	0 (0/156)^a^	0.94 (1/106)^a^	0.374	Fisher exact test
Sex hormone						
T (ng/mL), mean ± SE	3.78 ± 0.13^a^	3.83 ± 0.31^a^	3.56 ± 0.09^a^	3.92 ± 0.15^a^	0.309	one-way ANOVA test
LH (mIU/mL), mean ± SE	3.92 ± 0.21^a^	4.88 ± 1.22^a^	3.79 ± 0.15^a^	4.04 ± 0.21^a^	0.050	one-way ANOVA test
T/LH ratio, mean ± SE	1.20 ± 0.06^a^	1.12 ± 0.18^a^	1.10 ± 0.04^a^	1.20 ± 0.07^a^	0.571	one-way ANOVA test
FSH (mIU/mL), mean ± SE	6.21 ± 0.57^a^	8.78 ± 1.72^b^	5.85 ± 0.23^a^	5.75 ± 0.36^a^	–	one-way ANOVA test
E2 (pg/mL), mean ± SE	32.53 ± 1.52^a^	32.20 ± 4.14^a^	35.51 ± 1.08^a^	33.24 ± 1.30^a^	0.386	one-way ANOVA test
PRL (ng/mL), mean ± SE	10.40 ± 0.43^a^	13.49 ± 1.34^b^	10.06 ± 0.31^a^	11.56 ± 0.57^ab^	–	one-way ANOVA test

BMI, body mass index; T, testosterone; LH, luteinizing hormone; FSH, follicle‐stimulating hormone; E2, estradiol; PRL, prolactin; –, not applicable.

^a,b^Values without a common letter in their superscripts differ significantly (P < 0.05).

Sex hormonal analysis revealed that the levels of most hormones were similar among the unvaccinated, first-dose, second-dose, and booster vaccinated groups. Specifically, no significant differences were observed in T levels between the four groups (3.78 ± 0.13 vs. 3.83 ± 0.31 vs. 3.56 ± 0.09 vs. 3.92 ± 0.15 ng/mL). The serum LH levels showed no significant changes in patients who received any dose of inactivated SARS-CoV-2 vaccine compared to controls (3.92 ± 0.21 vs. 4.88 ± 1.22 vs. 3.79 ± 0.15 vs. 4.04 ± 0.21 mIU/mL). Moreover, we did not observe significant differences when T/LH ratios were compared (1.20 ± 0.06 vs. 1.12 ± 0.18 vs. 1.10 ± 0.04 vs. 1.20 ± 0.07). The serum E2 concentrations were also comparable between all groups (32.53 ± 1.52 vs. 32.20 ± 4.14 vs. 35.51 ± 1.08 vs. 33.24 ± 1.30 pg/mL). However, the FSH and PRL showed a highly significant increase in the first-dose vaccinated group, possibly due to the small sample size (n = 11).

**Table 2** showed the comparison of sperm parameters of the participants before and after vaccination. According to current data, vaccination with any single dose did not affect semen volume, sperm concentration, total sperm count, progressive motility, total motility, and normal forms. Most parameters of participants who received a combined dose of vaccine also yielded similar results. Nevertheless, compared to before vaccination, the total motility decreased substantially after fully vaccination (58.62 ± 2.51% vs. 46.90 ± 2.40%; *P* = 0.001). Fortunately, this parameter was still above the lower reference limit of WHO 5th edition [40% (38–42%)] (22). In addition, the average normal sperm morphologies of participants in both the control and vaccinated groups were below the recommended threshold of 4%. This may be due to the abstract criteria for sperm morphology defects, making it difficult for evaluators to give an accurate assessment. Moreover, the considerable heterogeneity in preparation, fixation, staining and smear reading techniques also has a serious impact on the sperm morphology evaluation results.

**Table 2:** Comparison of semen parameters before and after each dose or combination of doses of inactivated SARS-CoV-2 vaccine.

Parameter	Group A1		*P* value	Group B1		*P* value	Group C1		*P* value	Group D1		*P* value	Group E1		*P* value	Group F1		*P* value	WHO reference 5th percentile (median)
	Before 1st dose of vaccine	After 1st dose of vaccine		Before 2nd dose of vaccine	After 2nd dose of vaccine		Before booster dose of vaccine	After booster dose of vaccine		Before 1 + 2 doses of vaccine	After 1 + 2 doses of vaccine		Before 2 + 3 doses of vaccine	After 2 + 3 doses of vaccine		Before 1 + 2 + 3 doses of vaccine	After 1 + 2 + 3 doses of vaccine		
Participants’ Characteristic																			
Age (years), mean ± SE	28.67 ± 1.33			32.10 ± 1.74			32.13 ± 0.70			31.38 ± 0.62			30.71 ± 1.27			31.93 ± 0.82			
BMI (kg/m^2^), mean ± SE	27.20 ± 3.74			24.82 ± 1.60			27.38 ± 0.71			26.96 ± 0.50			25.23 ± 1.06			25.92 ± 0.69			
Semen parameter																			
Time from vaccination to semen sample collection (days), mean ± SE	–	12.00 ± 8.00	–	–	43.80 ± 17.90	–	–	61.58 ± 9.19	–	–	107.30 ± 7.72	–	–	52.29 ± 19.01	–	–	75.64 ± 10.28	–	–
Sexual abstinence (days), mean ± SE	3.67 ± 1.67	4.67 ± 1.20	0.654	4.40 ± 0.56	3.70 ± 0.34	0.302	4.00 ± 0.31	4.00 ± 0.32	1.000	4.25 ± 0.25	4.21 ± 0.25	0.919	4.29 ± 0.75	3.00 ± 0.38	0.159	3.79 ± 0.33	3.57 ± 0.31	0.641	2-7
Semen volume (ml), mean ± SE	3.67 ± 0.44	4.37 ± 0.45	0.328	3.56 ± 0.52	3.28 ± 0.60	0.728	3.69 ± 0.28	3.77 ± 0.29	0.834	3.89 ± 0.20	3.79 ± 0.23	0.743	4.04 ± 0.55	3.83 ± 0.73	0.818	3.60 ± 0.30	3.54 ± 0.30	0.892	1.5
Sperm concentration (10^6^ per ml), mean ± SE	25.43 ± 1.14	20.10 ± 3.91	0.305	46.45 ± 13.96	41.30 ± 13.77	0.796	25.62 ± 3.22	33.10 ± 4.50	0.181	33.06 ± 2.89	34.71 ± 3.62	0.723	23.80 ± 5.52	26.93 ± 9.02	0.773	27.67 ± 4.12	33.79 ± 4.94	0.345	15
Total sperm count (10^6^ per ejaculate), mean ± SE	94.04 ± 15.24	86.60 ± 17.64	0.766	155.08 ± 53.08	89.97 ± 22.51	0.281	90.03 ± 11.84	118.27 ± 18.23	0.198	130.42 ± 15.92	127.63 ± 17.70	0.907	99.69 ± 31.96	94.26 ± 31.85	0.906	90.99 ± 10.67	122.93 ± 20.61	0.176	39
Progressive motility (%), mean ± SE	34.70 ± 7.20	38.68 ± 6.30	0.700	29.88 ± 5.17	35.65 ± 5.42	0.451	31.50 ± 2.80	36.77 ± 3.08	0.210	38.99 ± 2.06	35.99 ± 2.07	0.304	29.28 ± 4.16	36.19 ± 5.58	0.342	35.41 ± 2.68	42.56 ± 2.87	0.074	32
Total motility (%), mean ± SE	49.96 ± 8.90	56.11 ± 9.42	0.660	54.28 ± 8.92	53.68 ± 8.55	0.961	42.49 ± 3.38	45.61 ± 3.53	0.526	58.62 ± 2.51	46.90 ± 2.40	0.001	48.15 ± 7.21	46.23 ± 7.49	0.856	52.45 ± 3.31	54.23 ± 3.54	0.715	40
Normal forms (%), mean ± SE	3.08 ± 0.03	3.10 ± 0.04	0.688	3.35 ± 0.34	3.60 ± 0.52	0.698	3.22 ± 0.16	3.27 ± 0.16	0.839	3.33 ± 0.10	3.22 ± 0.12	0.502	3.38 ± 0.39	3.68 ± 0.27	0.533	3.40 ± 0.12	3.63 ± 0.24	0.398	4

BMI, body mass index; –, not applicable; WHO, World Health Organization.

## 4 Discussion

In this study, we comprehensively investigated potential adverse effects of inactivated COVID-19 vaccination on male fertility. Compared to other studies (14, 19, 23), our study included booster dose and combinations of different doses of vaccines. We found that there were no changes in most semen parameters in males before and after receiving any dose or doses. Moreover, no significant differences in sex hormone levels were observed between vaccinated and unvaccinated subjects.

Sperm parameter analysis is viewed as one of the most fundamental and significant tools for assessing male fertility. In a study carried out by Edimiris et al., no significant short-term changes in ejaculate parameters were reported before and after viral vector vaccination (24). Likewise, Karavani et al. found that the mRNA vaccine did not negatively affect any of the sperm parameters over a relatively long-time period of 6–14 months from vaccination, even in men with male factor infertility (25). However, in our study, a slight decrease was demonstrated in sperm total motility after completion of two doses of the vaccine when compared to samples obtained before vaccination (group D1 in **Table 2**). The particularity with semen analysis was that we expected differences between the same individual providing two samples regardless of any intervention or treatment. There were also factors related to medical staff performance and the collection of the whole ejaculate by participants. More importantly, although the total motility decreased after the second vaccination, it remained higher than the WHO 5th percentile (46.90% *vs*. 40%) (22). Therefore, it is reasonable to infer that vaccination is safe with no apparent deleterious effect on semen parameters.

In fact, most of the concerns about the vaccine causing damage to male fertility stem from reports of the impact of COVID-19 on the reproductive system (14). Patients with severe, mild, or even asymptomatic COVID-19 were found to have abnormal semen parameters, as indicated by a decrease in semen volume, sperm concentration, total motility and progressive motility (26–31). It is worth noting that the total sperm count after a 90-day recovery period was still significantly below that of healthy controls (32). In addition to the effect on semen parameters, COVID-19 is associated with other male reproductive complications, such as the development of orchitis and erectile dysfunction (33, 34). Almost 10% to 23% of men diagnosed with acute SARS-CoV-2 infection developed orchitis or epididymo-orchitis (33, 35, 36). Notably, a cross-sectional study found that the prevalence of epididymitis was still as high as 42.3% among mild to moderate COVID-19 patients without testicular complaints (37). The histopathological findings showed Sertoli cell swelling and detachment, disfunction or reduction of Leydig cells, inflammatory infiltrations and accumulation of immunoglobulin G (29, 38, 39). In addition, Duarte-Neto et al. reported other testicular histological changes including vascular changes (endothelial edema, thrombosis), thickening of the tubular basal membrane and decreased spermatogenesis (40). Compared to pre-COVID-19 period, there were significant decreases in the International Index of Erectile Function score, sexual desire, intercourse satisfaction, orgasmic function, and frequency of sexual intercourse of the patients (41). Moreover, some of the recovered patients at 80 days were still diagnosed as erectile dysfunction along with psychological distress (42). The levels of sex hormones are also affected by SARS-CoV-2 infection. The SARS-CoV-2 nucleocapsid (N) protein, which has 90% amino acid homology with SARS-CoV N protein, is highly likely to be recognized by human immune responses (43). In an experimental study, Carrasco et al. found that the serum T levels in rats inoculated with the isolated N protein were significantly lower than those in the control group (44). Compared to healthy men, COVID-19 patients were accompanied by elevated serum LH levels and reduced T/LH ratios, suggesting possible subclinical impairment of male gonadal function (45, 46). These injuries could be attributed to several biological mechanisms. First, SARS-CoV-2 infection could induce the up-regulation of pro-inflammatory cytokine (e.g. IL-1b, IL-6 and TNF-α) and down-regulation of junctional proteins (e.g. occludin, claudin and connexin-43), resulting in a disruption of blood-testis barrier (47). Second, fever has been reported as one of the major symptoms in patients with COVID-19 (48, 49). In a meta-analysis of 67 studies and 8302 patients, above-normal body temperature was observed in 69% of positive cases (50). As we all know, temperature is crucial to the growth and development of cells. Under normal circumstances, the ideal temperature of sperm survival cannot be higher than 35°C, which is 2°C below the normal body temperature (51). When the human body is in a prolonged state of high fever, changes in testicular temperature occur, causing spermatogenesis to be impaired (52, 53). Third, a study of oxidative and antioxidative parameters of the seminal plasma suggested that infection significantly augmented oxidative stress (OS) responses and impaired antioxidant defense machinery, leading to enhanced reactive oxygen species (ROS) production and decreased levels of superoxide dismutase activity (54). At normal physiological levels, ROS mediates essential physiological mechanisms such as sperm maturation, capacitation, as well as fertilization (55). However, its excessive production and subsequent OS in male gonads have been proven to cause lipid peroxidation of sperm membranes and intracellular oxidative damage to spermatozoa, especially sperm DNA fragmentation (55, 56).

As mentioned earlier, the inactivated vaccine is produced in African green monkey kidney cells (Vero cells) that have been inoculated with SARS-CoV-2, then chemically inactivated using β-propiolactone and finally absorbed onto aluminum hydroxide (57). Thus, the degree of inflammation caused by it is much less than that results from infection. For example, a study confirmed elevated levels of both pro- and anti-inflammatory cytokines (including IFN-γ, IL-1ra, IL-10, IL-18, MCP-3, M-CSF and G-CSF) in COVID-19 patients, while no changes in various cytokines (including T helper 2 cell-related cytokines IL-4, IL-5, and IL-10) or lymphocyte subset distribution before and after vaccination were observed (58, 59). In addition, there are concerns that adverse reactions to inactivated vaccine may also affect fertility, especially fever. Fortunately, unlike the higher fever rate caused by infection, the percentage after vaccination did not exceed 5% in several evaluations. Most of the participants reported mild or moderate fever that was transient or resolved in few days (58, 60–62). Therefore, it is easy to understand that inactivated SARS-CoV-2 vaccination has no effect on sperm parameters.

This study has several limitations. First, the data from all participants were collected in a single center, thereby limiting the diversity of individuals studied. Second, this study did not evaluate male reproductive potential, i.e. comparing the results of assisted reproductive technologies cycles before and after vaccination. Third, the majority of population included in our study was men planning to conceive, which limited the generalizability of the conclusions.

## 5 Conclusion

Although there have been several studies on the impact of a single dose of inactivated SARS-CoV-2 vaccine on male reproductive capacity, this is the first time to comprehensively evaluate the effects of combination of different doses. Most sex hormone values remained unchanged after vaccination. However, the FSH and PRL levels in the one-dose vaccinated group were higher than other groups which may relate to its small sample size. The majority of semen parameters also showed similar results. Although the total motility decreased following two-dose vaccination compared to before vaccination, it was still within normal range. Accordingly, we are confident in suggesting that vaccination is safe with no adverse effect on male reproductive ability. Our result can be used to serve as a consulting tool for clinical doctors and reassure men who have already vaccinated to conceive. Future multicenter studies should be conducted to verify the generalizability of the research results and continue to monitor the long-term effects of vaccines on male health and sexual function.

## Data availability statement

The raw data supporting the conclusions of this article will be made available by the authors, without undue reservation.

## Ethics statement

The studies involving humans were approved by Ethics Committee of the Affiliated Hospital of Jining Medical University. The studies were conducted in accordance with the local legislation and institutional requirements. The participants provided their written informed consent to participate in this study.

## Author contributions

YD: Conceptualization, Formal analysis, Investigation, Methodology, Writing – original draft, Writing – review & editing. ZB: Conceptualization, Formal analysis, Investigation, Methodology, Writing – original draft, Writing – review & editing. YQ: Writing – original draft, Writing – review & editing. JM: Writing – original draft, Writing – review & editing. YL: Writing – original draft, Writing – review & editing. YZ: Writing – original draft, Writing – review & editing. AY: Conceptualization, Formal analysis, Investigation, Methodology, Writing – original draft, Writing – review & editing. FC: Conceptualization, Formal analysis, Funding acquisition, Investigation, Methodology, Writing – original draft, Writing – review & editing.

## References

[1] World Health Organization. Who Covid-19 Dashboard (2024). Available online at: https://covid19.who.int/.

[2] Li LQ Huang T Wang YQ Wang ZP Liang Y Huang TB. Covid-19 patients' Clinical characteristics, discharge rate, and fatality rate of meta-analysis. J Med Virol (2020) 92:577–83. doi: 10.1002/jmv.25757 PMC7228329 32162702

[3] Anderson RM Heesterbeek H Klinkenberg D Hollingsworth TD. How will country-based mitigation measures influence the course of the covid-19 epidemic? Lancet (2020) 395:931–4. doi: 10.1016/S0140-6736(20)30567-5 PMC7158572 32164834

[4] Lopez Bernal J Andrews N Gower C Robertson C Stowe J Tessier E. Effectiveness of the pfizer-biontech and oxford-astrazeneca vaccines on covid-19 related symptoms, hospital admissions, and mortality in older adults in England: test negative case-control study. Bmj (2021) 373:n1088. doi: 10.1136/bmj.n1088 33985964 PMC8116636

[5] World Health Organization. Weekly Epidemiological Update on Covid-19 - 18 May 2023 (2023). Available online at: https://www.who.int/publications/m/item/weekly-epidemiological-update-on-covid-19—18-may-2023.

[6] Mahase E. Covid-19: what do we know about xbb.1.5 and should we be worried? BMJ (2023) 380:153. doi: 10.1136/bmj.p153 36657748

[7] European Centre for Disease Prevention and Control. Threat assessment brief: Implications for the Eu/Eea of the spread of the SARS-CoV-2 Omicron Xbb.1.5 sub-lineage. (2023). Available at: https://www.ecdc.europa.eu/en/publications-data/covid-19-threat-assessment-brief-implications-spread-omicron-xbb.

[8] World Health Organization. Covid-19 Vaccine Tracker and Landscape (2023). Available online at: https://www.who.int/publications/m/item/draft-landscape-of-covid-19-candidate-vaccines.

[9] COVID-19 Vaccine Tracker. Approved Vaccines (2023). Available online at: https://covid19.trackvaccines.org/vaccines/approved/#vaccine-list.

[10] Lim WW Mak L Leung GM Cowling BJ Peiris M. Comparative immunogenicity of mrna and inactivated vaccines against covid-19. Lancet Microbe (2021) 2:e423. doi: 10.1016/S2666-5247(21)00177-4 34308395 PMC8282488

[11] Palacios R Batista AP Albuquerque CSN Patiño EG Santos J Conde MTRP. Efficacy and Safety of a Covid-19 Inactivated Vaccine in Healthcare Professionals in Brazil: The Profiscov Study (2021). Available online at: https://ssrn.com/abstract=3822780.

[12] Al Kaabi N Zhang Y Xia S Yang Y Al Qahtani MM Abdulrazzaq N. Effect of 2 inactivated sars-cov-2 vaccines on symptomatic covid-19 infection in adults: A randomized clinical trial. JAMA (2021) 326:35–45. doi: 10.1001/jama.2021.8565 34037666 PMC8156175

[13] Kang M Yi Y Li Y Sun L Deng A Hu T. Effectiveness of inactivated covid-19 vaccines against illness caused by the B.1.617.2 (Delta) variant during an outbreak in Guangdong, China : A cohort study. Ann Intern Med (2022) 175:533–40. doi: 10.7326/M21-3509 PMC8819853 35099990

[14] Zhu H Wang X Zhang F Zhu Y Du MR Tao ZW. Evaluation of inactivated covid-19 vaccine on semen parameters in reproductive-age males: A retrospective cohort study. Asian J Androl (2022) 24:441–4. doi: 10.4103/aja202225 PMC9491047 35532560

[15] Dong Y Li X Li Z Zhu Y Wei Z He J. Effects of inactivated sars-cov-2 vaccination on male fertility: A retrospective cohort study. J Med Virol (2023) 95:e28329. doi: 10.1002/jmv.28329 36415120

[16] Gat I Kedem A Dviri M Umanski A Levi M Hourvitz A. Covid-19 vaccination bnt162b2 temporarily impairs semen concentration and total motile count among semen donors. Andrology (2022) 10:1016–22. doi: 10.1111/andr.13209 PMC9350322 35713410

[17] Abd ZH Muter SA Saeed RAM Ammar O. Effects of covid-19 vaccination on different semen parameters. Basic Clin Androl (2022) 32:13. doi: 10.1186/s12610-022-00163-x 35915409 PMC9343088

[18] Xia W Zhao J Hu Y Fang L Wu S. Investigate the effect of covid-19 inactivated vaccine on sperm parameters and embryo quality in in vitro fertilization. Andrologia (2022) 54:e14483. doi: 10.1111/and.14483 35610731 PMC9348065

[19] Lestari SW Restiansyah G Yunihastuti E Pratama G. Comparison of sperm parameters and DNA fragmentation index between infertile men with infection and vaccines of covid-19. Asian J Androl (2023) 25:578–82. doi: 10.4103/aja202310 PMC10521957 37102509

[20] Andersson AM Jørgensen N Frydelund-Larsen L Rajpert-De Meyts E Skakkebaek NE. Impaired leydig cell function in infertile men: A study of 357 idiopathic infertile men and 318 proven fertile controls. J Clin Endocrinol Metab (2004) 89:3161–7. doi: 10.1210/jc.2003-031786 15240588

[21] Rohayem J Fricke R Czeloth K Mallidis C Wistuba J Krallmann C. Age and markers of leydig cell function, but not of sertoli cell function predict the success of sperm retrieval in adolescents and adults with klinefelter's syndrome. Andrology (2015) 3:868–75. doi: 10.1111/andr.12067 26235799

[22] World Health Organization. Who Laboratory Manual for the Examination and Processing of Human Semen Fifth Edition. Geneva: World Health Organization (2010).

[23] Huang J Xia L Tian L Xu D Fang Z Lin J. Comparison of Semen Quality before and after Inactivated Sars-Cov-2 Vaccination among Men in China. JAMA Netw Open (2022) 5:e2230631. doi: 10.1001/jamanetworkopen.2022.30631 36074470 PMC9459660

[24] Edimiris P Doehmen C Mueller L Andree M Baston-Buest DM Buest S. Vaccination with either Mrna or vector-based covid-19 vaccine has no detectable effect on sperm parameters. J Biomed Res Environ Sci (2022) 3:1076–81. doi: 10.37871/jbres1558

[25] Karavani G Chill HH Meirman C Gutman-Ido E Herzberg S Tzipora T. Sperm quality is not affected by the Bnt162b2 mrna sars-cov-2 vaccine: results of a 6-14 months follow-up. J Assist Reprod Genet (2022) 39:2249–54. doi: 10.1007/s10815-022-02621-x PMC9483282 36114906

[26] Che BW Chen P Yu Y Li W Huang T Zhang WJ. Effects of mild/asymptomatic covid-19 on semen parameters and sex-related hormone levels in men: A systematic review and meta-analysis. Asian J Androl (2023) 25:382–8. doi: 10.4103/aja202250 PMC10226500 35946226

[27] Hamarat MB Ozkent MS Yilmaz B Aksanyar SY Karabacak K. Effect of sars-cov-2 infection on semen parameters. Can Urol Assoc J (2022) 16:E173–E7. doi: 10.5489/cuaj.7292 PMC8923891 34672932

[28] Koc E Keseroglu BB. Does covid-19 worsen the semen parameters? Early results of a tertiary healthcare center. Urol Int (2021) 105:743–8. doi: 10.1159/000517276 PMC8339034 34265771

[29] Li H Xiao X Zhang J Zafar MI Wu C Long Y. Impaired spermatogenesis in covid-19 patients. EClinicalMedicine (2020) 28:100604. doi: 10.1016/j.eclinm.2020.100604 33134901 PMC7584442

[30] Best JC Kuchakulla M Khodamoradi K Lima TFN Frech FS Achua J. Evaluation of sars-cov-2 in human semen and effect on total sperm number: A prospective observational study. World J Mens Health (2021) 39:489–95. doi: 10.5534/wjmh.200192 PMC8255403 33663031

[31] Enikeev D Taratkin M Morozov A Petov V Korolev D Shpikina A. Prospective two-arm study of the testicular function in patients with covid-19. Andrology (2022) 10:1047–56. doi: 10.1111/andr.13159 PMC9111462 35124885

[32] Hu B Liu K Ruan Y Wei X Wu Y Feng H. Evaluation of mid- and long-term impact of covid-19 on male fertility through evaluating semen parameters. Transl Androl Urol (2022) 11:159–67. doi: 10.21037/tau-21-922 PMC8899150 35280660

[33] Pan F Xiao X Guo J Song Y Li H Patel DP. No evidence of severe acute respiratory syndrome-coronavirus 2 in semen of males recovering from coronavirus disease 2019. Fertil Steril (2020) 113:1135–9. doi: 10.1016/j.fertnstert.2020.04.024 PMC7164916 32482249

[34] Hsieh TC Edwards NC Bhattacharyya SK Nitschelm KD Burnett AL. The epidemic of covid-19-related erectile dysfunction: A scoping review and health care perspective. Sex Med Rev (2022) 10:286–310. doi: 10.1016/j.sxmr.2021.09.002 34732316 PMC8450276

[35] Chen L Huang X Yi Z Deng Q Jiang N Feng C. Ultrasound imaging findings of acute testicular infection in patients with coronavirus disease 2019: A single-center-based study in Wuhan, China. J Ultrasound Med (2021) 40:1787–94. doi: 10.1002/jum.15558 33174632

[36] Ediz C Tavukcu HH Akan S Kizilkan YE Alcin A Oz K. Is there any association of COVID-19 with testicular pain and epididymo-orchitis? Int J Clin Pract (2021) 75:e13753. doi: 10.1111/ijcp.13753. 33063899 PMC7646040

[37] Carneiro F Teixeira TA Bernardes FS Pereira MS Milani G Duarte-Neto AN. Radiological patterns of incidental epididymitis in mild-to-moderate covid-19 patients revealed by colour doppler ultrasound. Andrologia (2021) 53:e13973. doi: 10.1111/and.13973 33565141 PMC7994978

[38] Yang M Chen S Huang B Zhong JM Su H Chen YJ. Pathological findings in the testes of covid-19 patients: clinical implications. Eur Urol Focus (2020) 6:1124–9. doi: 10.1016/j.euf.2020.05.009 PMC7261470 32563676

[39] Nie X Qian L Sun R Huang B Dong X Xiao Q. Multi-organ proteomic landscape of covid-19 autopsies. Cell (2021) 184:775–91 e14. doi: 10.1016/j.cell.2021.01.004 33503446 PMC7794601

[40] Duarte-Neto AN Teixeira TA Caldini EG Kanamura CT Gomes-Gouvêa MS Dos Santos ABG. Testicular pathology in fatal covid-19: A descriptive autopsy study. Andrology (2022) 10:13–23. doi: 10.1111/andr.13073 34196475 PMC8444746

[41] Sevim M Alkis O Kartal IG Telli S Aras B. A factor not to be ignored in post-covid-19 erectile dysfunction; psychological effect, a prospective study. Androl (2022) 54:e14443. doi: 10.1111/and.14443 PMC9111346 35445425

[42] Hu B Ruan Y Liu K Wei X Wu Y Feng H. A mid-to-long term comprehensive evaluation of psychological distress and erectile function in covid-19 recovered patients. J Sex Med (2021) 18:1863–71. doi: 10.1016/j.jsxm.2021.08.010 PMC8387224 34600862

[43] Grifoni A Sidney J Zhang Y Scheuermann RH Peters B Sette A. A sequence homology and bioinformatic approach can predict candidate targets for immune responses to sars-cov-2. Cell Host Microbe (2020) 27:671–80.e2. doi: 10.1016/j.chom.2020.03.002 32183941 PMC7142693

[44] Lucio Carrasco CH Noda P Barbosa AP Vieira Borges da Silva EK Gasque Bomfim C Ventura Fernandes BH. Sars-cov-2 nucleocapsid protein is associated with lower testosterone levels: an experimental study. Front Physiol (2022) 13:867444. doi: 10.3389/fphys.2022.867444 35721551 PMC9204174

[45] Kharbach Y Khallouk A. Male genital damage in covid-19 patients: are available data relevant? Asian J Urol (2021) 8:324–6. doi: 10.1016/j.ajur.2020.06.005 PMC7306201 32837913

[46] Ma L Xie W Li D Shi L Ye G Mao Y. Evaluation of sex-related hormones and semen characteristics in reproductive-aged male covid-19 patients. J Med Virol (2021) 93:456–62. doi: 10.1002/jmv.26259 PMC7361404 32621617

[47] Peirouvi T Aliaghaei A Eslami Farsani B Ziaeipour S Ebrahimi V Forozesh M. Covid-19 disrupts the blood-testis barrier through the induction of inflammatory cytokines and disruption of junctional proteins. Inflammation Res (2021) 70:1165–75. doi: 10.1007/s00011-021-01497-4 PMC8387554 34436630

[48] Bassi A Henry BM Pighi L Leone L Lippi G. Evaluation of indoor hospital acclimatization of body temperature before covid-19 fever screening. J Hosp Infect (2021) 112:127–8. doi: 10.1016/j.jhin.2021.02.020 PMC7906508 33640369

[49] Navarra A Albani E Castellano S Arruzzolo L Levi-Setti PE. Coronavirus disease-19 infection: implications on male fertility and reproduction. Front Physiol (2020) 11:574761. doi: 10.3389/fphys.2020.574761 33312128 PMC7704452

[50] Mair M Singhavi H Pai A Singhavi J Gandhi P Conboy P. A meta-analysis of 67 studies with presenting symptoms and laboratory tests of covid-19 patients. Laryngoscope (2021) 131:1254–65. doi: 10.1002/lary.29207 33068023

[51] He Y Wang J Ren J Zhao Y Chen J Chen X. Effect of covid-19 on male reproductive system - a systematic review. Front Endocrinol (Lausanne) (2021) 12:677701. doi: 10.3389/fendo.2021.677701 34122351 PMC8190708

[52] Xu J Qi L Chi X Yang J Wei X Gong E. Orchitis: A complication of severe acute respiratory syndrome (Sars). Biol Reprod (2006) 74:410–6. doi: 10.1095/biolreprod.105.044776 PMC7109827 16237152

[53] Carlsen E Andersson AM Petersen JH Skakkebaek NE. History of febrile illness and variation in semen quality. Hum Reprod (2003) 18:2089–92. doi: 10.1093/humrep/deg412 14507826

[54] Hajizadeh Maleki B Tartibian B. Covid-19 and male reproductive function: A prospective, longitudinal cohort study. Reproduction (2021) 161:319–31. doi: 10.1530/rep-20-0382 33522983

[55] Dutta S Majzoub A Agarwal A. Oxidative stress and sperm function: A systematic review on evaluation and management. Arab J Urol (2019) 17:87–97. doi: 10.1080/2090598x.2019.1599624 31285919 PMC6600059

[56] Sengupta P Dutta S. Does sars-cov-2 infection cause sperm DNA fragmentation? Possible link with oxidative stress. Eur J Contracept Reprod Health Care (2020) 25:405–6. doi: 10.1080/13625187.2020.1787376 32643968

[57] Hassan W Kazmi SK Tahir MJ Ullah I Royan HA Fahriani M. Global acceptance and hesitancy of covid-19 vaccination: A narrative review. Narra J (2021) 1:e57. doi: 10.52225/narra.v1i3.57 38450215 PMC10914054

[58] Xia S Duan K Zhang Y Zhao D Zhang H Xie Z. Effect of an inactivated vaccine against sars-cov-2 on safety and immunogenicity outcomes: interim analysis of 2 randomized clinical trials. JAMA (2020) 324:951–60. doi: 10.1001/jama.2020.15543 PMC7426884 32789505

[59] Yang Y Shen C Li J Yuan J Wei J Huang F. Plasma IP-10 and MCP-3 levels are highly associated with disease severity and predict the progression of COVID-19. J Allergy Clin Immunol (2020) 146:119–27.e4. doi: 10.1016/j.jaci.2020.04.027 PMC7189843 32360286

[60] Han B Song Y Li C Yang W Ma Q Jiang Z. Safety, tolerability, and immunogenicity of an inactivated sars-cov-2 vaccine (Coronavac) in healthy children and adolescents: A double-blind, randomised, controlled, phase 1/2 clinical trial. Lancet Infect Dis (2021) 21:1645–53. doi: 10.1016/S1473-3099(21)00319-4 PMC8238449 34197764

[61] Zhang MX Zhang TT Shi GF Cheng FM Zheng YM Tung TH. Safety of an inactivated sars-cov-2 vaccine among healthcare workers in China. Expert Rev Vaccines (2021) 20:891–8. doi: 10.1080/14760584.2021.1925112 PMC8127168 33929930

[62] Xia S Zhang Y Wang Y Wang H Yang Y Gao GF. Safety and immunogenicity of an inactivated sars-cov-2 vaccine, bbibp-corv: A randomised, double-blind, placebo-controlled, phase 1/2 trial. Lancet Infect Dis (2021) 21:39–51. doi: 10.1016/s1473-3099(20)30831-8 33069281 PMC7561304

